# Assessing Fatty Infiltration of Paraspinal Muscles in Patients With Lumbar Spinal Stenosis: Goutallier Classification and Quantitative MRI Measurements

**DOI:** 10.3389/fneur.2021.656487

**Published:** 2021-09-03

**Authors:** Filippo Mandelli, Corina Nüesch, Yuancheng Zhang, Florian Halbeisen, Stefan Schären, Annegret Mündermann, Cordula Netzer

**Affiliations:** ^1^Department of Spine Surgery, University Hospital Basel, Basel, Switzerland; ^2^Department of Orthopaedics, University Children's Hospital Basel, Basel, Switzerland; ^3^Department of Biomedical Engineering, University of Basel, Basel, Switzerland; ^4^Department of Clinical Research, University of Basel, Basel, Switzerland; ^5^Department of Orthopaedics and Traumatology, University Hospital Basel, Basel, Switzerland; ^6^Institute for Clinical Epidemiology and Biostatistics, University Hospital Basel, Basel, Switzerland

**Keywords:** paraspinal muscles fatty infiltration, muscle fatty infiltration, Goutallier, paraspinal cross-sectional area, paraspinal muscles morphology

## Abstract

**Objective:** Fatty infiltration of paraspinal muscle is associated with spinal disorders. It can be assessed qualitatively (i.e., Goutallier classification) and quantitatively using image processing software. The aims of this study were to compare paraspinal muscle fatty infiltration as assessed using the Goutallier classification vs. quantitative magnetic resonance images (MRI) measurements and to investigate the association between anthropometric parameters and paraspinal muscle morphology and fatty infiltration in patients with symptomatic lumbar spinal stenosis (LSS).

**Methods:** Patients affected by symptomatic LSS scheduled for surgery with available MRI of the lumbar spine were included in this retrospective cross-sectional study. Fatty infiltration at each lumbar level was rated qualitatively according to the Goutallier classification and quantified based on the cross-sectional area (CSA) of the paraspinal muscle, of its lean fraction (LeanCSA), and the ratio between LeanCSA and CSA and the CSA relative to the CSA of vertebral body (RCSA). Considering the muscle as a single unit, overall fatty infiltration according to Goutallier, overall CSA, LeanCSA, LeanCSA/CSA, and RCSA were computed as averages (aGoutallier, aCSA, aLeanCSA, aLeanCSA/aCSA, and aRCSA). Associations among parameters were assessed using Spearman's respective Pearson's correlation coefficients.

**Results:** Eighteen patients, with a mean age of 71.3 years, were included. aGoutallier correlated strongly with aLeanCSA and aLeanCSA/aCSA (*R* = −0.673 and *R* = −0.754, both *P* < 0.001). There was a very strong correlation between values of the left and right sides for CSA (*R* = 0.956, *P* < 0.001), LeanCSA (*R* = 0.900, *P* < 0.001), and LeanCSA/CSA (*R* = 0.827, *P* < 0.001) at all levels. Among all anthropometric measurements, paraspinal muscle CSA correlated the most with height (left: *R* = 0.737, *P* < 0.001; right: *R* = 0.700, *P* < 0.001), while there was a moderate correlation between vertebral body CSA and paraspinal muscle CSA (left: *R* = 0.448, *P* < 0.001; right: *R* = 0.454, *P* < 0.001). Paraspinal muscle CSA correlated moderately with body mass index (BMI; left: *R* = 0.423, *P* < 0.001; right: *R* = 0.436, *P* < 0.001), and there was no significant correlation between aLeanCSA or aLeanCSA/CSA and BMI.

**Conclusions:** The Goutallier classification is a reliable yet efficient tool for assessing fatty infiltration of paraspinal muscles in patients with symptomatic LSS. We suggest taking body height as a reference for normalization in future studies assessing paraspinal muscle atrophy and fatty infiltration.

## Introduction

Muscle impairment is an important component of spinal disorders. In fact, muscle dysfunction can be considered both a cause and a consequence of low back pain (LBP) (1)—for instance, several studies have shown an association between fatty infiltration and the reduced cross-sectional area (CSA) of paraspinal muscles with chronic LBP (2–7). Moreover, fatty degeneration may lead to functional limitations such as deterioration of balance and alignment of the spine (8, 9). However, to date, the contribution of compromised muscles to pain in spinal pathologies, such as facet arthropathy, disc degeneration, spinal stenosis, or deformity, is unclear. Paraspinal muscle fatty degeneration and atrophy have been reported in patients with LBP and disc herniation (10–13) or degenerative lumbar flat back (14). In patients with disc herniation, muscle infiltration may be asymmetric and more pronounced on one side than the other (15). Moreover, greater fatty infiltration in muscles such as multifidus, longissimus, and psoas correlates with poorer functional outcomes with an even stronger relation in persons with a history of LBP (16). Similarly, patients with lumbar spinal stenosis (LSS) or symptomatic LSS (sLSS) show increased muscle atrophy and fatty infiltration (17). LSS is one of the most frequent spinal disorders and the most common reason for spine surgery in the population aged above 65 years (18). While there are several studies investigating the correlation of muscle composition and morphology in patients with LBP, to date, evidence for this relationship in patients with LSS is lacking.

Fatty degeneration of the multifidus and reduced CSA of the psoas muscles have been associated with a lower functional performance in terms of higher scores on the Oswestry Disability Index (ODI) (19–21). In addition, a reduced CSA of the multifidus may predispose patients to worse outcome after surgery for sLSS (22). Moreover, the relation between CSA of paraspinal muscles and anthropometric parameters has been explored. The results of previous studies are inconclusive, where some studies did not observe a significant association between paraspinal muscle CSA and body height or body mass (23–25), while others reported a greater paraspinal CSA in taller and heavier persons (26).

Magnetic resonance imaging (MRI) facilitates the detailed investigation of the lumbar spine and the estimation of the morphology and composition of paraspinal muscles, including multifidus and erector spinae (longissimus and iliocostalis). These assessments can be performed in a qualitative or in a quantitative way. The Goutallier classification is a visual grading system to qualitatively assess fatty infiltration (27–30). Initially proposed for grading fatty degeneration of the rotator cuff muscles on computer tomography, the Goutallier classification has been expanded to MRI and to the evaluation of other muscles, including back muscles (6, 31, 32). Moreover, there is evidence of positive correlations between Goutallier grades and the severity of disc degeneration as well as age (33). Previous studies have shown a substantial to excellent intraobserver and a good interobserver reliability (32, 33) as well as a significant positive correlation of Goutallier grades, with the percentage of fat in the multifidus muscle measured in a quantitative way (32). Although the Goutallier classification is simple to apply and a useful tool for clinicians when evaluating their patients, it has the disadvantages of qualitative measurements and ordinal scales. This classification depends on the experience of the assessor and is reported on an ordinal scale with five discrete levels. In contrast, the quantitative MRI measurements of paraspinal muscles overcome such limitations by being objective and continuous measures. However, these quantitative measures are more time consuming and hence more suitable for research rather than clinical settings. To date, data on comparisons between qualitative and quantitative assessments of paraspinal muscle fatty infiltration and their association with patient characteristics in patients with sLSS are lacking. The aims of this study were to compare paraspinal muscle fatty infiltration as assessed using the Goutallier classification vs. quantitative MRI measurements, assess asymmetry in muscle degeneration, and investigate the association between anthropometric parameters and paraspinal muscle morphology and fatty infiltration in patients with sLSS.

## Materials and Methods

This single center cross-sectional study was approved by the regional ethics committee and conducted in accordance with the Declaration of Helsinki. All enrolled patients provided written informed consent.

### Study Cohort

Patients with a diagnosis of sLSS scheduled for decompressive surgery at the University Hospital Basel from April 2019 to August 2020 were screened for this study. The participants were recruited in the context of a larger clinical research project, and eligible patients were informed about the study after admission to the hospital on the preoperative day. The inclusion criteria were as follows: diagnosed sLSS and availability of MR images of the lumbar spine from L1 to S1. The exclusion criteria were the following: prior surgery of the lumbar spine, additional pathologies that influence the mobility of the pelvis (such as internal fixation of the sacro-iliac joint or hip disorders affecting the gait), use of walking aids, and inability to provide informed consent. Age, sex, body mass, body height, and body mass index (BMI) were recorded. The level of LBP of the participants and the extent to which the pain impacts their daily activities and social life were estimated using the validated German version of the standardized questionnaire ODI (34, 35). The ODI comprises 10 self-administered items describing the pain and limitations experienced when performing daily activities: pain intensity, personal care, lifting, walking, sitting, standing, sleeping, sexual life, social life, and traveling. The resulting score ranges from 0 (no impact) to 100 (bed-bound and extremely limited).

### Lumbar MRI

All patients received MRI of the lumbar spine for clinical purposes. All MRI examinations included at least a sagittal T1- or T2-weighted sequence that was used to define the corresponding axial cut to be measured and an axial T2-weighted sequence to perform the qualitative and quantitative measurement of muscle morphology and composition. The MR images were obtained at our clinic (Prisma 3T, Siemens Healthineers, Erlangen, Germany) or provided by external providers at first consultation.

### Qualitative Assessment of Paraspinal Muscle Fatty Infiltration

**Figure 1:**
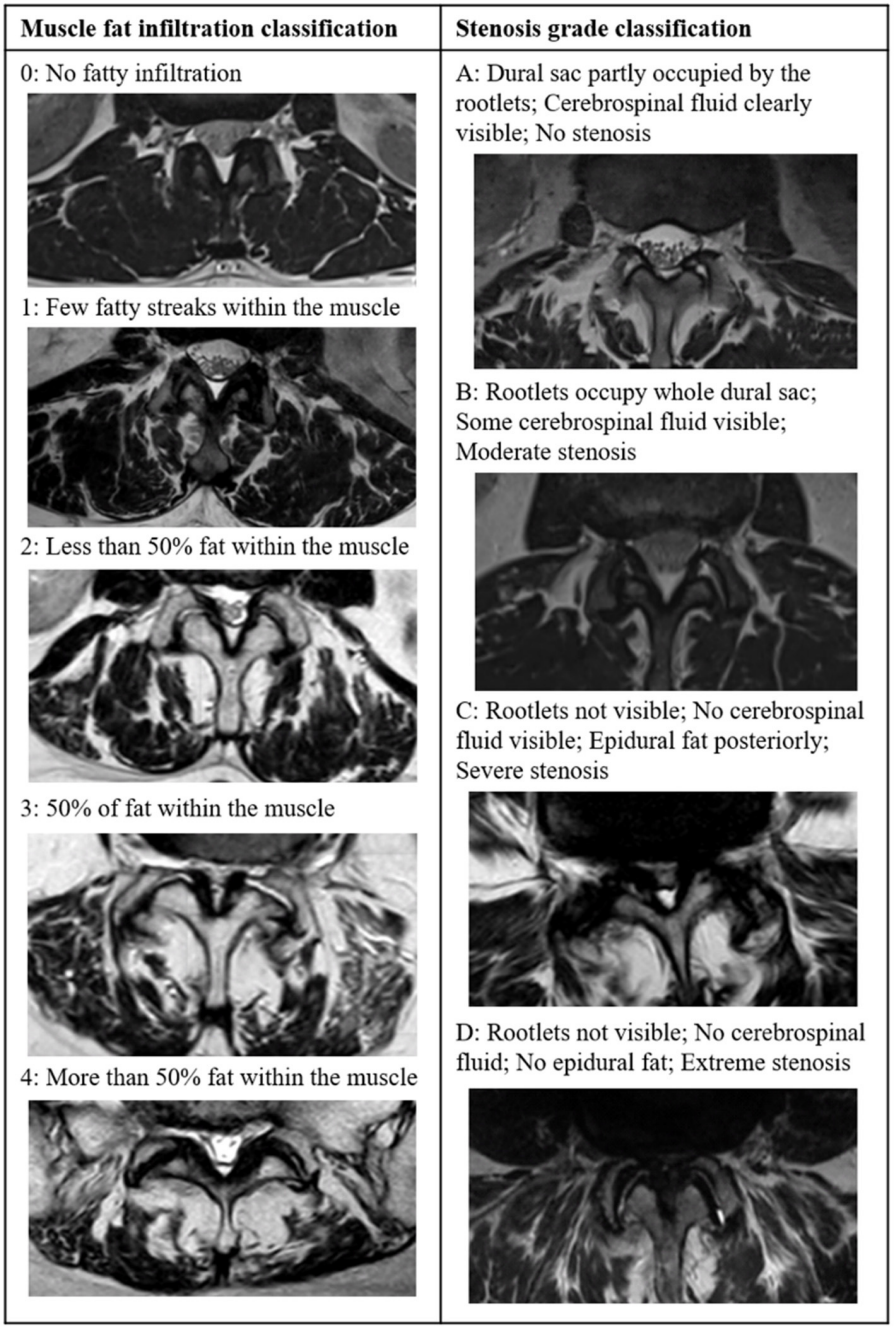
T2-weighted axial images showing the grading of fatty infiltration of paraspinal muscles according to the Goutallier classification (left) (27) and grading of lumbar spinal stenosis according to the Schizas classifications (right) (36).

A qualitative assessment of paraspinal muscle fatty infiltration was performed using the Goutallier classification system (27). Accordingly, the muscle composition of the paraspinal muscles multifidus and longissimus on MRI was classified independently by two readers (FM and YZ) into five different grades based on the visually assessed fat/muscle ratio at each disc level from L1/L2 to L5/S1 (five segments in total; Figure 1). The inter-reader reliability was 0.701 (Cohens Kappa, *P* < 0.001). In case of disagreement between assessments, consensus was reached by a third reader (CNe). The grades range from grade 0—no visible fatty infiltration to grade 4—more than 50% of fat within the muscle. Overall qualitative fatty infiltration was computed as average Goutallier (aGoutallier) of all segments because fatty infiltration measured at each segment presumably affects the function of the entire muscle.

### Quantitative Assessment of Paraspinal Muscle Fatty Infiltration

**Figure 2:**
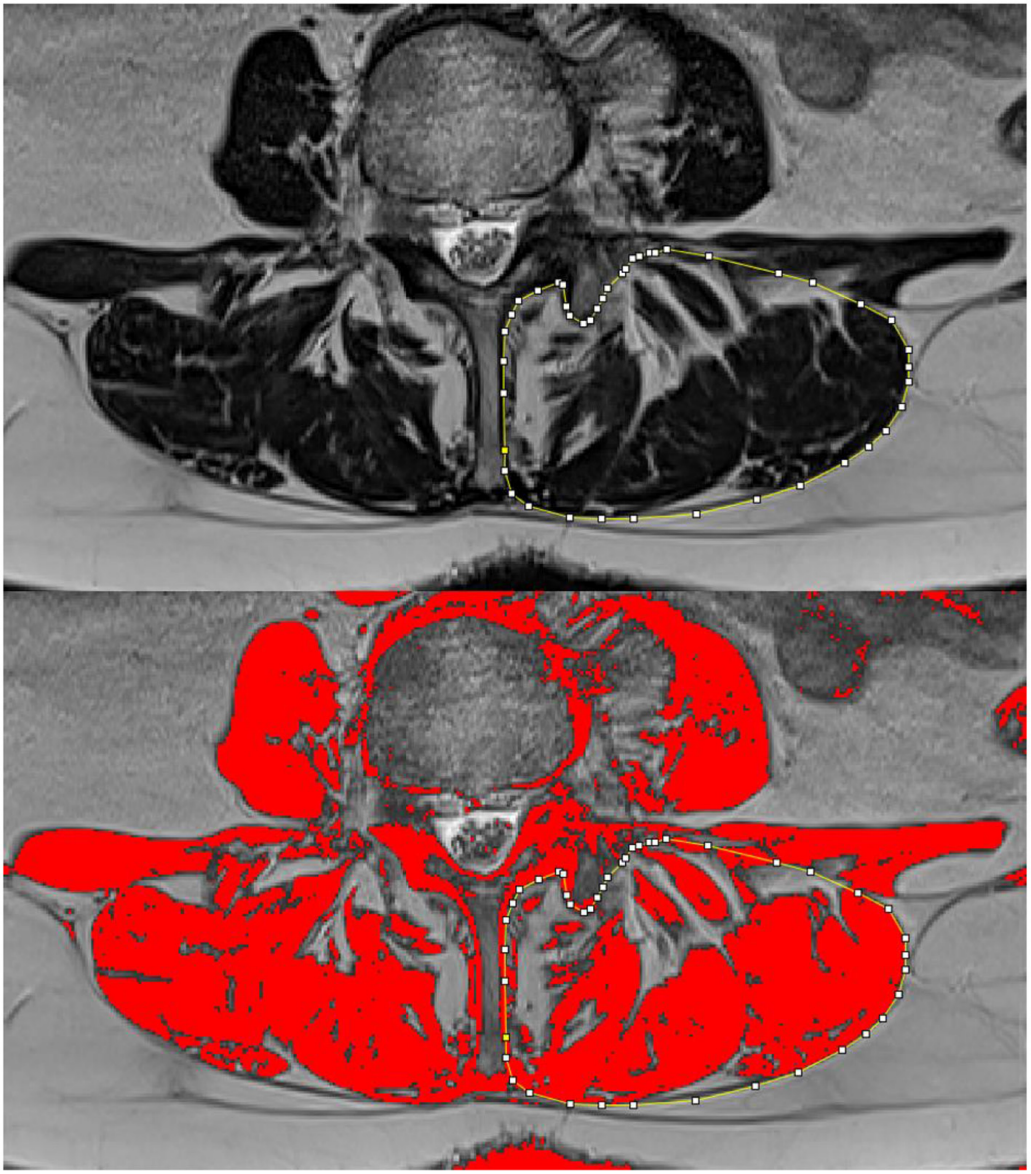
Using the ImageJ analysis software, the left paraspinal muscles are contoured and the cross-sectional area is measured (top); after thresholding, the lean muscle is represented in red, and its area (LeanCSA) is measured (bottom).

The quantitative assessment of paraspinal muscle fatty infiltration was performed using ImageJ image analysis software (version 1.52t, National Institutes of Health, Bethesda, Maryland) according to Fortin et al. (20, 37). ImageJ has been used in previous studies to assess fatty infiltration in MRI series of the lumbar spine (20, 32, 38, 39) and has shown a good intra- and interobserver reliability (37). Measurements of paraspinal muscle fatty infiltration were performed independently by two assessors (FM and YZ). A T2-weighted axial image was selected at each vertebral body level in the center of the body itself as identified on the sagittal-view image. For L1 to L5, we measured the CSA of the paraspinal muscles on each side, including the multifidus and the erector spinae (longissimus and iliocostalis) muscles, and the CSA of the vertebral body. The inter-reader reliability was very high [intraclass correlation coefficient, ICC (95% confidence interval)]—CSA: 0.939 (0.917, 0.955) and vertebral body CSA: 0.894 (0.722, 0.944). The relative CSA (RCSA) was defined as the ratio between muscle CSA and vertebral body CSA and calculated for each level and side. Thresholding of grayscale for lean muscle was repeated for each level, and each image was segmented according to the specific threshold (Figure 2). The CSA of lean muscle in the region of interest was defined as LeanCSA and measured on each side. The inter-reader reliability of LeanCSA was ICC (95% confidence interval)—LeanCSA: 0.959 (0.944, 0.970). The ratio of LeanCSA to the paraspinal muscle CSA was defined as functional CSA (LeanCSA/CSA), represented as percent of muscle CSA and calculated for each level and side. The overall CSA, RCSA, and LeanCSA were computed as average CSA (aCSA), average RCSA (aRCSA), and average LeanCSA (aLeanCSA) across all levels, considering the muscle as a single unit for each side. The total muscle CSA, total RCSA, and total LeanCSA at each level were calculated as the sum of the left and right muscle CSA, RCSA, and LeanCSA, respectively. Overall LeanCSA/CSA was computed as average LeanCSA/CSA (aLeanCSA/aCSA) of all segments and both sides.

### Radiological Assessment of Severity of Spinal Stenosis

We graded the severity of the spinal stenosis according to the Schizas classification (36). The grading system is based on cerebral spine fluid/rootlet ratio on axial T2 images and consists of seven grades (A1, A2, A3, A4, B, C, and D) of stenosis (Figure 2). The severity of stenosis was assessed for each lumbar level addressed during the scheduled surgery and carried out by one reader (FM). The number of levels with stenosis and the highest severity of stenosis were determined and used in the analysis. A1 to A4 grades were grouped into a single group A.

### Statistical Analysis

Statistical analyses were performed in SPSS Statistics, version 27 (IBM Corporation, Armonk, New York, USA). All data were checked for normality using the Kolmogorov–Smirnov test. The descriptive statistics for normally distributed parameters were performed using mean and one standard deviation (SD) and for all others as median and interquartile range (IQR). Differences in CSA, RCSA, LeanCSA, and LeanCSA/CSA between levels were identified for each side using analysis of variance (ANOVA) for repeated measures, with levels as within-subject factor, and upon significant results, *t*-tests for dependent samples were performed as *post-hoc* tests for comparisons between pairs of levels. The associations between continuous and normally distributed parameters were detected using Pearson's correlation coefficient. The associations between or with ordinal (Goutallier grade) and/or not normally distributed parameters were detected using Spearman's correlation coefficient. All correlations were performed separately for each side, except for correlations between parameters describing average muscle atrophy and fatty infiltration where the combined values for both sides were included. Correlations were considered very weak for 0 ≤ |*R*| < 0.2, weak for 0.2 ≤ |*R*| < 0.4, moderate for 0.4 ≤ |*R*| < 0.6, strong for 0.6 ≤ |*R*| < 0.8, and very strong for 0.8 ≤ |*R*| ≤ 1.0 (40). The significance level for all tests was set *a priori* to 0.05.

## Results

### Patient Characteristics

Eighteen patients with a mean age of 71.3 years (SD: 8.4) were included. The proportion of male patients was 45% (eight of 18 patients). The mean body mass was 75.8 kg (SD: 15.0). The mean height was 167.1 cm (SD: 8.6). The mean BMI of all patients was 27.0 kg/m^2^ (SD: 3.9). The mean ODI score was 28.7 (SD: 13.5). The highest severity of LSS across all levels was grade B in four (22.2%) patients, grade C in 12 (66.7%) patients, and grade D in two (11.1%) patients. None of the patients had the highest stenosis severity grade A.

### Qualitative Assessment of Paraspinal Muscle Fatty Infiltration

**Figure 3:**
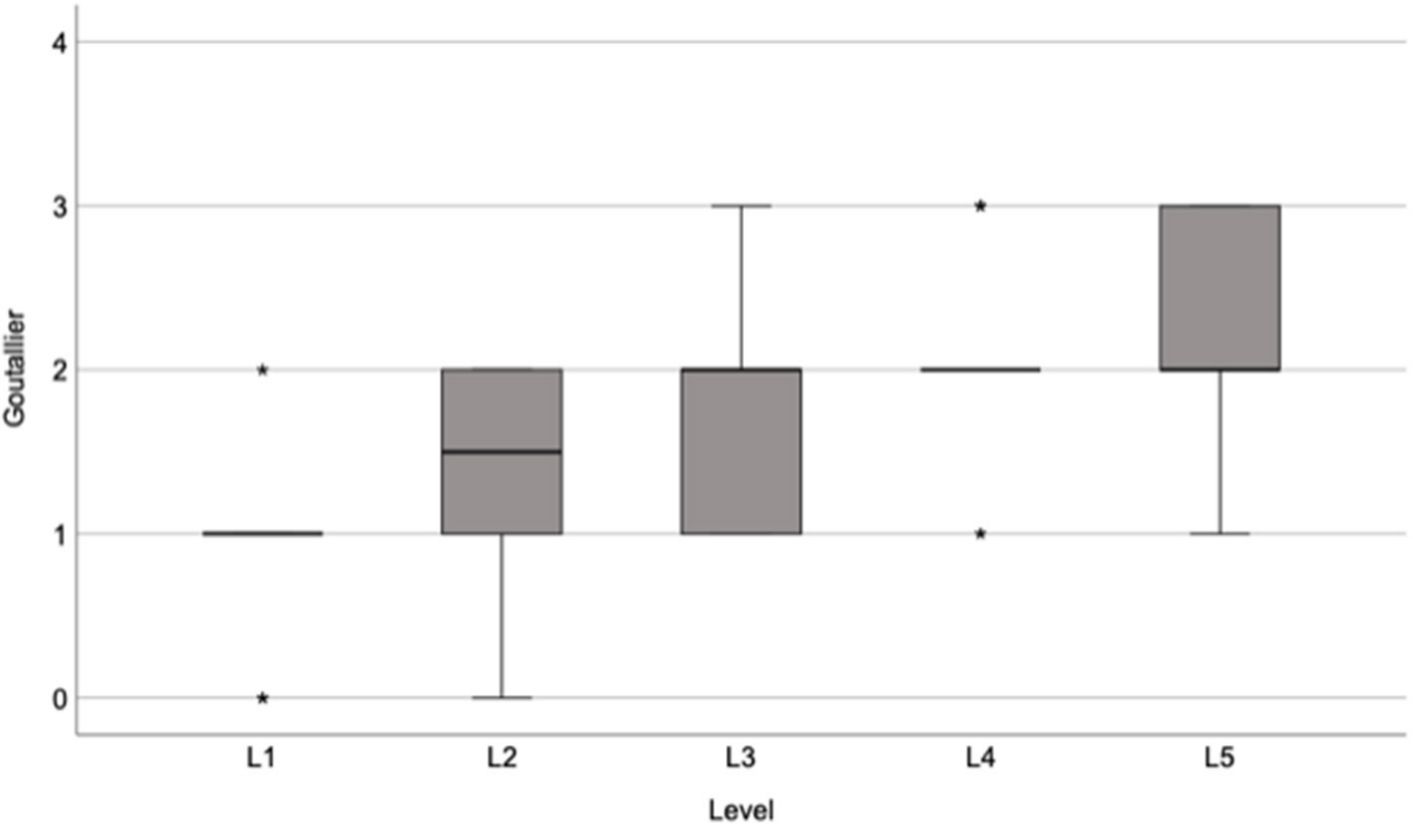
Box plot showing the median and interquartile range of Goutallier grades at each level of the lumbar spine.

The median fatty infiltration according to the Goutallier classification system among all levels in all patients was 2.0 (IQR: 1.0–3.0). Figure 3 shows the Goutallier grade at each level. The mean aGoutallier of all patients was 1.7 (SD: 0.6).

### Quantitative Assessment of Paraspinal Muscles

**Figure 4:**
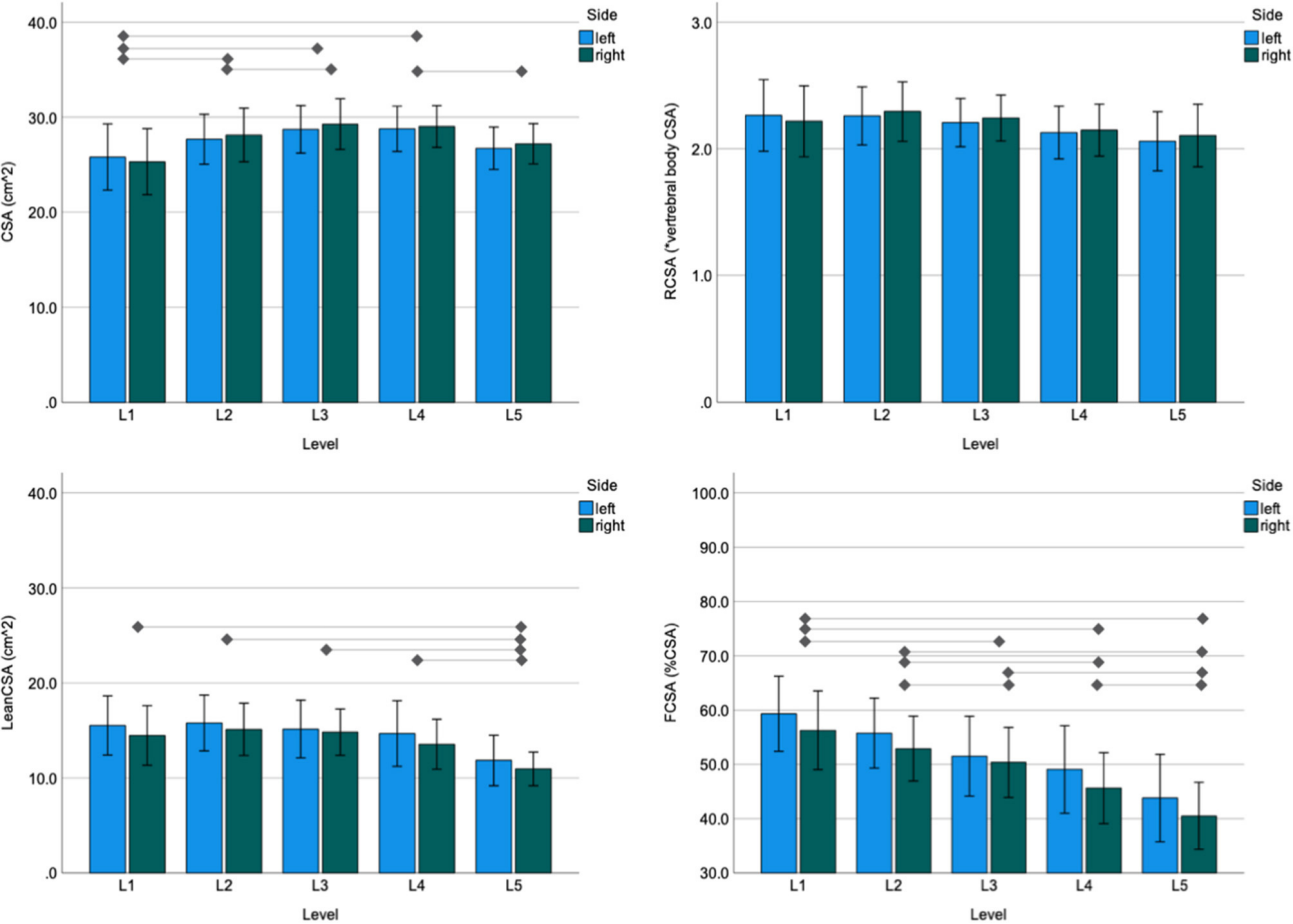
Mean and 95% confidence interval of the paraspinal cross-sectional area (CSA; top left), relative CSA (RCSA; top right), lean muscle cross-sectional area (LeanCSA; bottom left), and the ratio of LeanCSA/CSA and CSA (LeanCSA/CSA; bottom right) for the left and right sides at each level of the lumbar spine. The horizontal lines indicate significant differences between levels. Because significant results of paired comparisons were observed for both sides for each indicated pair, horizontal bars represent differences for both sides (*t*-test for paired samples, *P* < 0.05).

The MR images of two subjects did not include an axial cut of the L1 level; data at this level for 16 of the 18 patients were included. The sum of the left and right total aCSA of the paraspinal muscles was 54.2 cm^2^ (SD: 9.3), with the highest total paraspinal muscle CSA value of 58.0 cm^2^ (SD: 9.9) at the L3 level and the lowest value of 45.4 cm^2^ (SD: 20.0) at the L1 level (Figure 4). The statistically significant differences in paraspinal muscle CSA between levels are indicated in Figure 4. The total aRCSA was 4.4 (SD: 0.7), with the highest total (sum of left and right) RCSA value of 4.5 (SD: 0.7) at L2 level, although the differences between levels were not statistically significant (Figure 4). The total aLeanCSA was 27.7 cm^2^ (SD: 10.0), with the highest total (sum of left and right) LeanCSA value of 30.9 cm^2^ (SD: 10.9) at L2 and the lowest value of 22.8 cm^2^ (SD: 8.2) at L5 (Figure 4). The statistically significant differences in LeanCSA between levels are indicated in Figure 4. The total aLeanCSA/aCSA was 50.2% (SD: 12.0%), where the values decreased from L1 to L5, with the highest value of 57.8% (SD: 12.6%) at L1 and the lowest value of 42.2% (SD: 13.3%) at L5 (Figure 4). The statistically significant differences in LeanCSA/CSA between levels are indicated in Figure 4.

### Association Between Qualitative and Quantitative Measures of Fatty Infiltration

**Figure 5:**
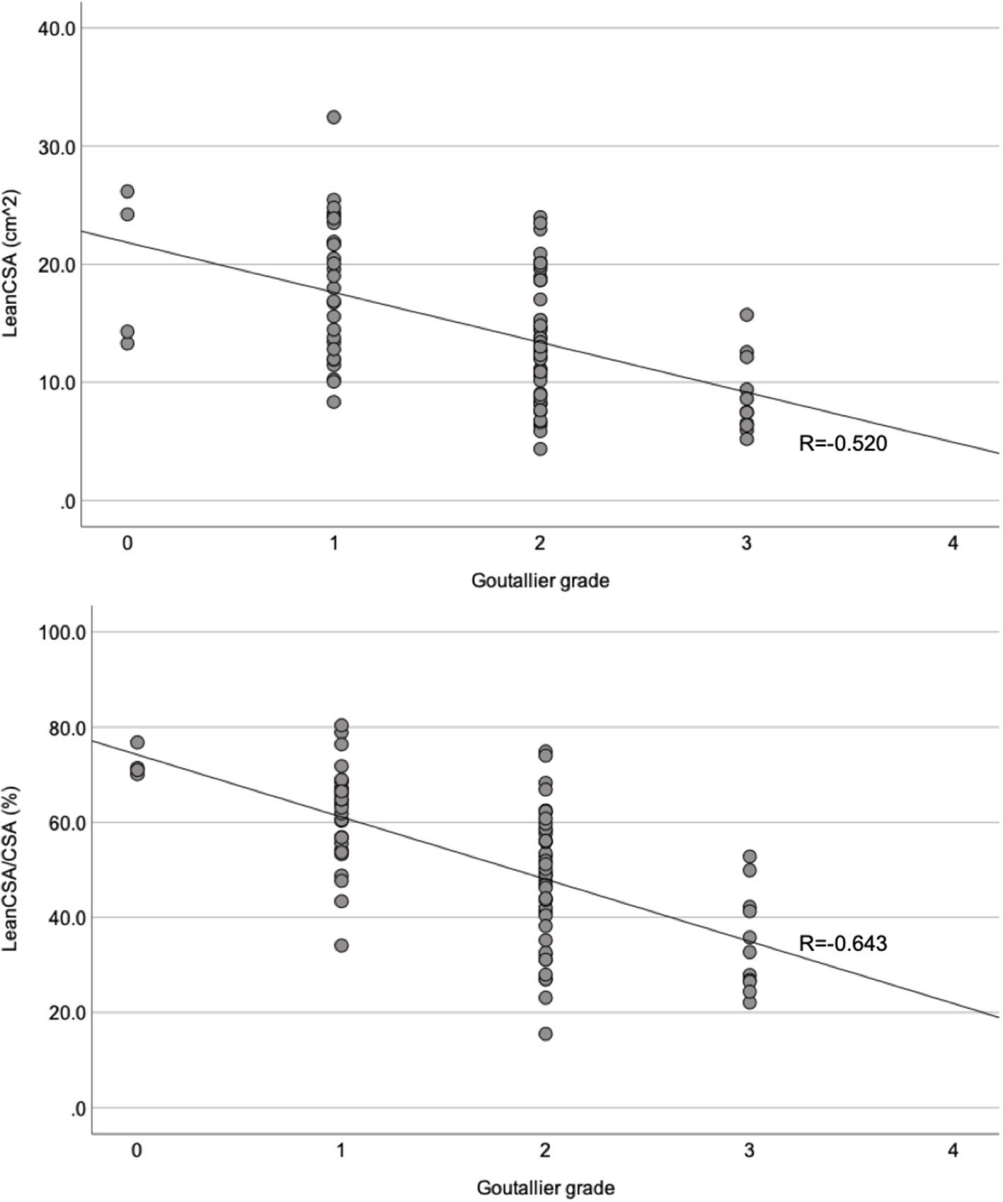
Scatter plots comparing the Goutallier grade for the paraspinal muscles with the lean muscle cross-sectional area (LeanCSA; top) and the ratio between LeanCSA and paraspinal muscle cross-sectional area (LeanCSA/CSA; bottom) of the left side. *R*, Spearman's correlation coefficient.

**Figure 6:**
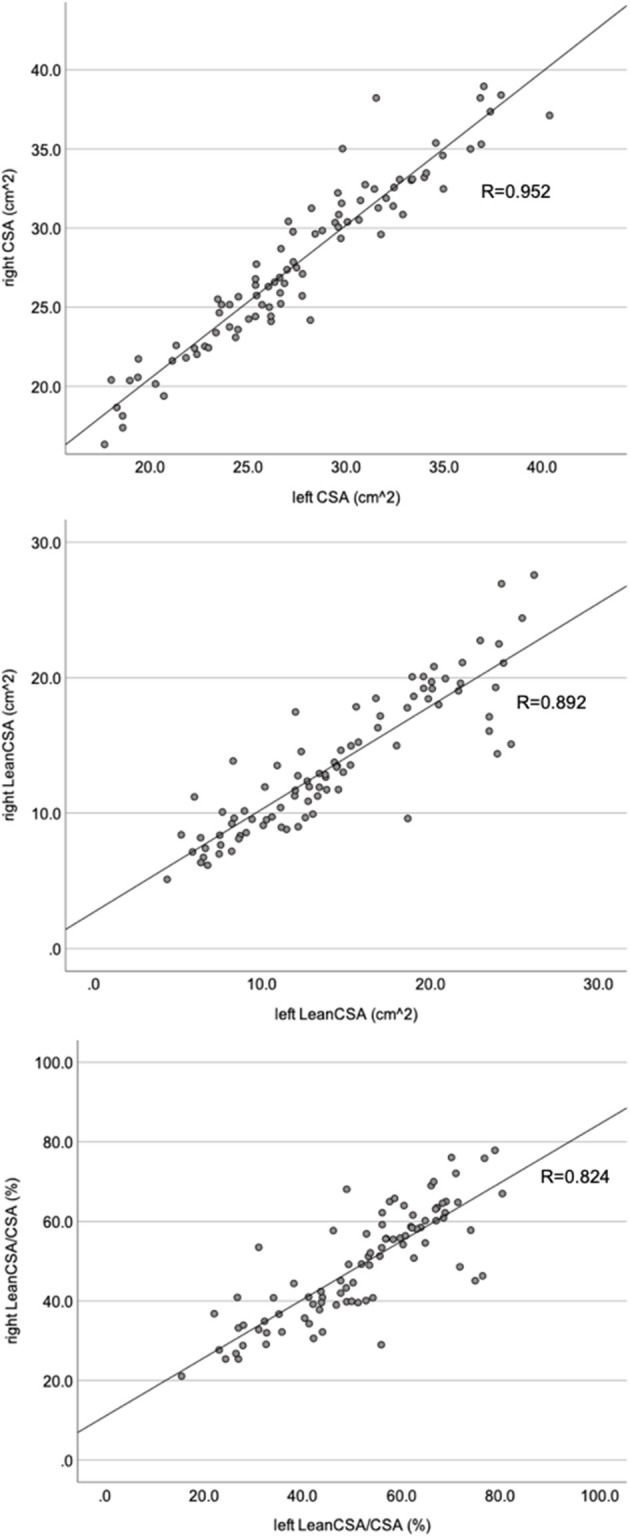
Scatter plots comparing the left and right sides of the paraspinal muscle cross-sectional area (CSA; top), lean muscle cross-sectional area (LeanCSA; middle) and the ratio between LeanCSA and the paraspinal muscle cross-sectional area (LeanCSA/CSA; bottom). *R*, Pearson's correlation coefficient.

There was a moderate correlation between the Goutallier classification system and LeanCSA across all levels, both for the left and right sides (left: *R* = −0.520, *P* < 0.001; right: *R* = −0.497, *P* < 0.001; Figure 5). There was a strong correlation between Goutallier grades and LeanCSA/CSA across all levels on both sides (left: *R* = −0.643, *P* < 0.001; right: *R* = −0.604, *P* < 0.001; Figure 5). Across both sides, aGoutallier correlated strongly with aLeanCSA and aLeanCSA/aCSA (*R* = −0.673 and *R* = −0.754, both *P* < 0.001) (Supplementary Figure 1). There was a very strong correlation between the values of the left and right sides for CSA (*R* = 0.956, *P* < 0.001), LeanCSA (*R* = 0.900, *P* < 0.001), and LeanCSA/CSA (*R* = 0.827, *P* < 0.001) considering all levels (Figure 6).

### Association Between MRI Measurements and Morphometric Parameters

There was a strong correlation between paraspinal muscle CSA and body height (left: *R* = 0.737, *P* < 0.001; right: *R* = 0.700, *P* < 0.001) and between LeanCSA and body height (left: *R* = 0.648, *P* < 0.001; right: *R* = 0.612, *P* < 0.001). There was a weak to moderate correlation between LeanCSA/CSA and body height (left: *R* = 0.442, *P* < 0.001; right: *R* = 0.340, *P* = 0.001). The Goutallier grade correlated weakly with body height (*R* = −0.219, *P* = 0.039). There was a moderate correlation between vertebral body CSA and body height (*R* = 0.536, *P* < 0.001). The paraspinal muscle CSA correlated moderately with the BMI (left: *R* = 0.423, *P* < 0.001; right: *R* = 0.436, *P* < 0.001), and there was no significant correlation between LeanCSA or LeanCSA/CSA and BMI. None of the parameters describing fatty infiltration correlated with age in this cohort.

We observed a moderate correlation between vertebral body CSA and paraspinal muscle CSA (left: *R* = 0.448, *P* < 0.001; right: *R* = 0.454, *P* < 0.001). The vertebral body CSA showed no significant correlation with LeanCSA or LeanCSA/CSA.

## Discussion

The aims of this study were to compare paraspinal muscle fatty infiltration as assessed using the Goutallier classification vs. quantitative MRI measurements and to investigate the association between anthropometric parameters and paraspinal muscle morphology and fatty infiltration in patients with sLSS. We observed a moderate to strong correlation between the Goutallier classification system of muscle fatty infiltration and the quantitative assessment of the LeanCSA and lean fraction of the paraspinal muscle CSA, named as LeanCSA/CSA. Moreover, paraspinal muscle morphology and fatty infiltration correlated strongly with body height. These results support the value of assessing fatty infiltration at all levels in the lumbar region and the importance of considering the stature of a patient when interpreting fatty infiltration in the context of LSS.

Patients with higher Goutallier grades had lower LeanCSA and LeanCSA/CSA, representing smaller absolute and relative lean muscle CSA. Similarly, Battaglia et al. reported a strong to very strong correlation for MR images of 25 randomly selected subjects between Goutallier grades and mean percent fat value measured with the same method as in our study (32). Though, to date, the role of fatty infiltration and atrophy of paraspinal muscles is not clear, there is evidence of their association with LBP—for instance, Kjaer et al. found that fatty infiltration of the multifidus muscle was strongly associated with LBP (5). These results are consistent with those of Goubert et al., who found a positive correlation of fatty infiltration with chronic LBP (4). Other authors investigated the association of muscle morphology and composition with spinal disorders. Yanik et al. reported a significant increase in fatty infiltration in individuals affected by disc herniation (6), and this was corroborated by other studies (15, 41). Yarjanian et al. found a reduced functional CSA of paraspinal muscles in patients with sLSS compared to asymptomatic individuals; however, the difference was not significant when compared to subjects with chronic LBP without evidence of LSS (17). Overall, the evidence for the relation of back muscle to spinal disorders emphasizes the importance for clinicians to regularly evaluate the morphology of paraspinal muscles on the MR images of patients. However, quantitatively measuring paraspinal muscle CSA and lean fraction is time consuming and hence not feasible in the clinical setting. Our results showed that fatty atrophy, assessed using the Goutallier classification system, strongly correlates with quantitative measures and thus—despite being a qualitative measure—is a good and, most importantly, efficient method for evaluating the degeneration of paraspinal muscles in sLSS.

The quantitative measures of paraspinal muscle morphology and fatty infiltration CSA, LeanCSA, and LeanCSA/CSA correlated strongly between the left and right sides across all levels of the lumbar spine. We interpret this as a sign of symmetry in muscle morphology and degeneration. However, because we did not record data on the laterality of symptoms nor of spinal stenosis, we cannot exclude that, in case of asymmetric symptoms or spinal stenosis, an asymmetric muscle degeneration may be observed. In fact, in a study on patients affected by disc herniation, Battié et al. reported greater fatty infiltration in the multifidus muscle on the side of the radicular compression at the level below the herniation, although the total paraspinal muscle CSA was greater on the affected side (15). In another study on patients with symptomatic posterolateral disc herniation at L4 and L5, Fortin et al. did not observe an asymmetry of the multifidus but of the erector spinae muscle, with smaller CSA and greater fatty infiltration on the affected side both of the multifidus muscle and the erector spinae muscle (41). The mechanism of these findings seems to be related to abnormal muscle activation as a result of the altered neural signal (42). Conversely, we did not observe any significant asymmetry of the paraspinal muscles. While we did not assess a possible laterality of symptoms and laterality, we would have only been able to detect a systematic laterality of all patients, which was not the scope of this study. Another possible explanation for this discrepancy is that LSS and its symptoms are more commonly bilateral compared to disc herniation. Nevertheless, further investigation is needed to support this statement.

Fatty infiltration generally increased from cranial to caudal, with the highest value at L5. This result confirms previous studies by Kjaer et al. (5) and Lee et al. (14), who showed that L4 and L5 are the segments most affected by fatty infiltration. In our study, both LeanCSA and LeanCSA/CSA differed significantly between L5 and all other levels. One possible explanation is that most degenerative spinal processes involve the segments L4/L5 and L5/S1 (43), but there is a lack of evidence regarding a causal link between fatty atrophy of paraspinal muscles and spinal disorders. Furthermore, Lee et al. (14) observed an increasing fatty infiltration toward the more caudal levels in 10 healthy volunteers. Kjaer et al. (5) found that the lowest lumbar levels had the greatest signs of fatty infiltration regardless of age in adults and adolescents with a history of LBP. These results suggest that the most caudal lumbar levels are physiologically more prone to fatty infiltration even in young and healthy individuals. Spinal pathologies known to be more frequent at these levels and to be associated with an increase in fatty infiltration of the back muscles may further exaggerate the difference in muscle composition between the cranial and caudal portions of the paraspinal muscles.

Our secondary aim was to determine the association between quantitative measurements of paraspinal muscle morphology and fatty infiltration and anthropometric parameters. We found that CSA and LeanCSA had a strong and LeanCSA/CSA had a weak correlation with body height. The results of previous studies on this association are inconclusive—for instance, our results confirm those reported by Gibbons et al., who observed a positive correlation between the CSA of paraspinal muscles and body height, body mass, and BMI in a large sample of male monozygotic twins (130 subjects) (26). In contrast, two smaller studies by Wood et al. (25) and McGill et al. (23) (13 and 26, respectively) did not find any significant association of CSA with body height, body mass, or BMI in persons with various suspected injuries and diseases. Moreover, Reid et al. reported that greater body height was not a predictor of larger CSA (24). Biomechanically, an association of CSA with body height is intuitive because the greater a person is, the greater are the moment arms of the forces generated by the weight of the upper torso, arms, and head relative to the lumbar spine area during daily activities. Frequently, the paraspinal total and lean CSA are normalized to the vertebral body or intervertebral disc CSA (19, 44–47). It is interesting to note that, in our study, both overall CSA and LeanCSA correlated more strongly with body height than with vertebral CSA (strong *vs*. moderate correlation). Although we consider the vertebral body or the disc as reasonable choice of reference for reducing the bias of differences in anthropometric measures between persons, our results suggest that body height greatly influences paraspinal muscle CSA and should hence be used for normalization.

In our study, only CSA, but not LeanCSA or LeanCSA/CSA, correlated with BMI. This result is consistent with the study by Kjaer et al. (5) who did not find BMI to have an influence on the amount of fatty infiltration in the paraspinal muscles. Moreover, Gibbons et al. (26) found that a greater BMI was a predictor of greater CSA, and Kalichman et al. (48) reported a negative correlation between muscle density on CT scan and BMI. Our results show that people with higher BMI are not prone to greater fatty infiltration of paraspinal muscles, possibly because stronger muscles are required to account for the larger body weight in obese persons when stabilizing the spine. These observations suggest that the higher levels of fatty infiltration of paraspinal muscles are likely not related to obesity but rather to the degenerative process of the muscle itself or to the spinal disorder affecting the patient.

The main strength of our study is the measurement of qualitative and quantitative parameters of paraspinal muscles at each level of the lumbar spine regardless of the level of stenosis. Despite the evidence that fatty infiltration is greater between L4 and S1 and given the anatomy and function of the erector spinae and multifidus muscles, we believe that these entire muscles should be considered as a single unit. Hence, we introduced global qualitative and quantitative parameters for assessing fatty atrophy of the entire paraspinal muscle unit which aGoutallier and aLeanCSA defined as the average of the respective parameters across all levels. The main limitation of our study is the small sample size, and thus we could not correct for multiple testing. However, while this study can be considered a proof-of-principle investigation, the strong correlations and clear differences observed in our study suggest that these results should hold true when investigated in larger samples. Moreover, we included only individuals affected by sLSS and scheduled for surgery. Similar analyses should be conducted in patients with less severe LSS or who were treated conservatively and in age-matched healthy controls to elucidate the role of muscle atrophy and fatty infiltration in the etiology of the disease and the effects of different treatments on these parameters. The MR images were either obtained at our clinic or transferred by external providers. While using the same make and model for all measurements may have provided even better results, we intended to conduct this study in a real-world environment. In other words, if the same make and model had been used for all patients, the generalizability of the data to other MRI systems would have been unknown/limited. In contrast, our data showed strong correlations despite the different make and models of the MRI systems used, suggesting that the agreement between the assessments is robust regarding the specific MRI system, making our result even more relevant in a clinical context. Finally, while the quantitative method employed here has shown good interobserver and excellent intraobserver reliability, the accuracy of the thresholding procedure has yet to be confirmed with MRI sequences based on chemical shift (Dixon) or spectroscopy. Nonetheless, the sequence used here is a standard clinical sequence, and extracting information on fatty infiltration in standard clinical sequences is relevant especially in cases where Dixon sequences are not available.

## Conclusion

The correlation of the Goutallier classification with the quantitative assessment of fatty infiltration of paraspinal muscles suggests that clinicians should consider this classification as an efficient tool for evaluating paraspinal muscle fatty infiltration. Nonetheless, this qualitative measure does not consider muscle morphology, which may add insight into the role that paraspinal muscle status plays in the etiology of LSS. Because paraspinal muscle CSA correlates with body height, we suggest taking body height as a reference for normalization in future studies assessing paraspinal muscle atrophy and fatty infiltration.

## Data Availability Statement

The raw data supporting the conclusions of this article will be made available by the authors, without undue reservation.

## Ethics Statement

The studies involving human participants were reviewed and approved by Ethikkommission Nordwest- und Zentralschweiz (EKNZ). The patients/participants provided their written informed consent to participate in this study.

## Author Contributions

FM: conceptual study design, data collection and processing, and manuscript writing. CNü: conceptual study design, patient recruitment, data collection and processing, statistical analysis, and manuscript review. YZ: data compilation and manuscript review. FH: statistical analysis and manuscript review. SS: conceptual study design and manuscript review. AM: conceptual study design, data analysis, statistical analysis, and manuscript writing and review. CNe: conceptual study design, patient recruitment, and manuscript review. All authors contributed to the article and approved the submitted version.

## Conflict of Interest

The authors declare that the research was conducted in the absence of any commercial or financial relationships that could be construed as a potential conflict of interest.

## Publisher's Note

All claims expressed in this article are solely those of the authors and do not necessarily represent those of their affiliated organizations, or those of the publisher, the editors and the reviewers. Any product that may be evaluated in this article, or claim that may be made by its manufacturer, is not guaranteed or endorsed by the publisher.

## Supplementary Material

Supplementary Figure 1 Scatter plots comparing the average Goutallier grade across all levels (aGoutallier) with the average lean muscle cross-sectional area (aLeanCSA; top) and the ratio between average LeanCSA and average paraspinal muscle cross-sectional area (aLeanCSA/aCSA; bottom). *R*, Pearson's correlation coefficient.
