# Supplementary figures and images for: Assessing Fatty Infiltration of Paraspinal Muscles in Patients With Lumbar Spinal Stenosis: Goutallier Classification and Quantitative MRI Measurements
Source: Front Neurol. 2021 Sep 3;12:656487. doi: 10.3389/fneur.2021.656487 (PMC8446197; doi:10.3389/fneur.2021.656487)

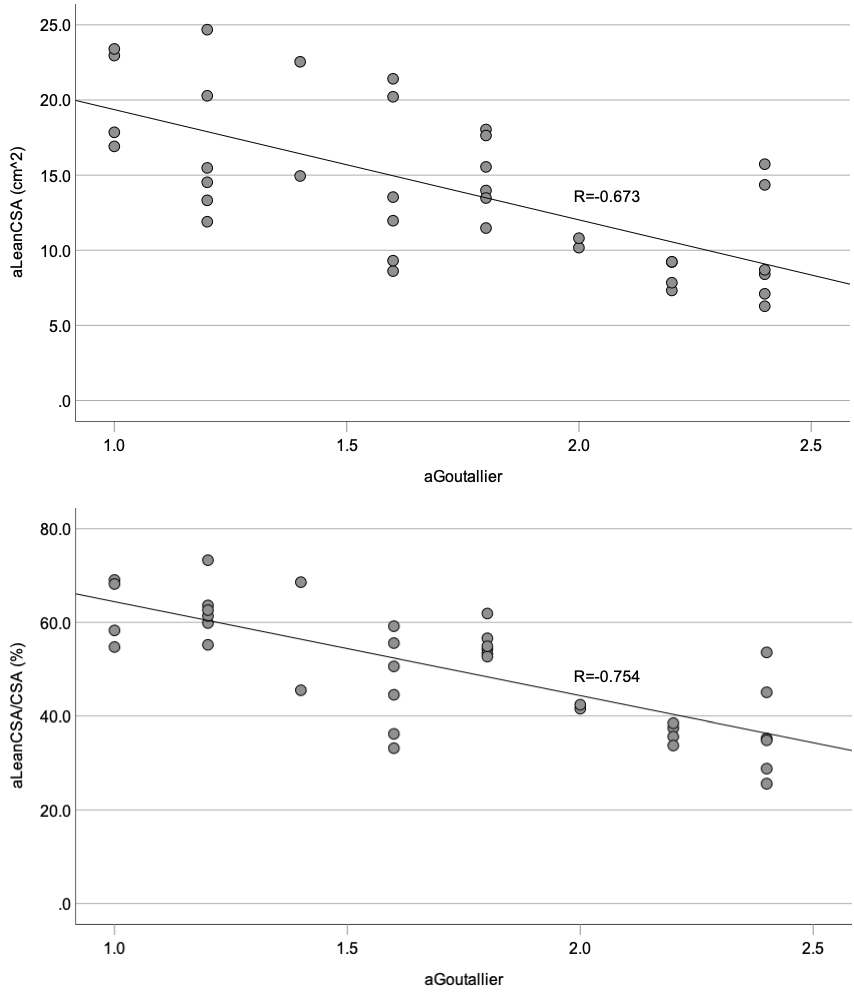

Supplement: Supplementary Figure 1 — Scatter plots comparing the average Goutallier grade across all levels (aGoutallier) with the average lean muscle cross-sectional area (aLeanCSA; top) and the ratio between average LeanCSA and average paraspinal muscle cross-sectional area (aLeanCSA/aCSA; bottom). R, Pearson's correlation coefficient. [file Image_1.TIFF]
